# An Unjustifiable Operation

**Published:** 1895-09

**Authors:** 


### AN UNJUSTIFIABLE OPERATION.

The desire of the surgeon to have his name go down to posterity attached to some discovery or operation has led to the introduction of some surgical procedures which, to use an Hibernicism, have been "an advance backwards." Perhaps it is not fair in every case to set down operations that have turned out badly to the inventor's desire for fame, but certainly many surgical procedures must have been recommended to the world by their originators before sufficient time and pains had been taken to find out just what was the value of the new thing. The cost of finding out must be borne principally by the patient, but also to no small extent by those who make the experiments.

The frequency with which hemorrhoids occur and the ease with which they may be cured has stimulated surgical inventors to find new operations for them. In consequence the surgeon may choose one of a round half dozen procedures for their relief, each of which has its staunch advocates; these procedures range in severity and in their radical nature from simple stretching of the sphincter, recommended and practiced by Verneuil and other French surgeons, to the operation of Whitehead, by which the lowest inch of the mucous membrane of the rectum is dissected up and removed. The self-styled "orificial surgeons" have adopted Whitehead's operation under the imposing title, "the American operation," or as it is often abbreviated, simply "the American," the only difference being that in Whitehead's operation the dissection is made from below upwards, while in "the American" it is made from above downwards. In each case the severed edge of mucous membrane above is pulled down and sutured to the skin at its junction with the mucous membrane at the anus, the point where the lower of the two circular incisions is made.

Prof. Edmund Andrews, of Chicago, in a recent paper strongly condemns the mutilation of the lower part of the rectum in the manner just described, on the ground that the piece of mucous membrane removed plays an important part in the act of defectation and in the retention of fæces and gases in the lower bowel. This inch of mucous membrane contains the

*columnæ Morgagni*, a number of short vertical folds with shallow sulci between, each sulcus ending below in a little pouch known as the *sacculi Horneri;* below the columns are a number of small papillæ, each of which, according to Andrews, contains a tactile nerve corpuscle endowed with an acute special sense, and is a part of the reflex mechanism by which the sphincter ani muscle is kept tightly contracted whenever fæces or gases present at the outlet of the bowel. This reflex action keeps the bowel closed without the necessity of the intervention of the will power, by which also the contraction of the sphincter may be maintained. The sulci and pouches contain a viscid mucus which serves to lubricate the fæces and aid their expulsion. These anatomical structures have been rediscovered by the orificial humbugs and pronounced to be pathological lesions, which, under the names of "pockets" and "papillæ" have been slit up and snipped off respectively in thousands of cases for the cure of an infinite variety of diseases of which they were the alleged source. The Whitehead and "American" operations, by sweeping away these structures have often badly crippled the rectum in the performance of important functions.

Prof. Andrews has obtained from leading specialists in rectal surgery in this country and abroad a list of two hundred and one cases where evil results have followed the operation in question. Of these bad effects the one oftenest noted is, as might be supposed, incontinence of fæces and gases, a most deplorable condition for the patient. Moreover, of the surgeons corresponded with, at least four-fifths were strongly opposed to the operation, and of those who favored it at all, the most limited its applicability to a few selected cases. Yet the orificial quacks are performing it all the time, with the result that Kelsey says the misfits of the "American operation" are a constant source of income to him, ten per cent of the patients, he thinks, needing a second operation to cure them of the bad results of the first. If the Whithead operation produces no better results than this, the best thing would be for the regular profession to drop it altogether, and then to unite in condemning its counterfeit, "the American."—*Northwestern Lancet.*

